# [Retracted] Triptolide inhibits migration and proliferation of fibroblasts from ileocolonic anastomosis of patients with Crohn’s disease via regulating the miR‑16‑1/HSP70 pathway

**DOI:** 10.3892/mmr.2025.13549

**Published:** 2025-04-28

**Authors:** Min Chen, Jin-Min Wang, Dong Wang, Rong Wu, Hong-Wei Hou

Mol Med Rep 19: 4841–4851, 2019; DOI: 10.3892/mmr.2019.10117

Following the publication of the above article, a concerned reader drew to the authors' attention that, regarding the western blot data shown in Fig. 1, the Col-I protein bands were strikingly similar to the bands that were featured in the third and fourth lanes of Fig. 5G to represent the α-SMA data. In addition, the protein bands shown for the Col-I and α-SMA experiments in the first and second lanes of the respective gels in Fig. 5G also appeared to be strikingly similar.

Upon examining these data independently in the Editorial Office, the Editor of *Molecular Medicine Reports* has determined that this article should be retracted from the Journal on the basis of a lack of confidence in the presented data. Upon contacting the authors, they accepted the decision to retract this article. The Editor apologizes to the readership of the Journal for any inconvenience caused.

